# Deregulation of dicer and mir-155 expression in liposarcoma

**DOI:** 10.18632/oncotarget.3201

**Published:** 2015-04-04

**Authors:** Bruno Vincenzi, Michele Iuliani, Alice Zoccoli, Francesco Pantano, Marco Fioramonti, Delia De Lisi, Anna Maria Frezza, Carla Rabitti, Giuseppe Perrone, Andrea Onetti Muda, Antonio Russo, Antonio Giordano, Daniele Santini, Angelo Paolo Dei Tos, Giuseppe Tonini

**Affiliations:** ^1^ Medical Oncology, Campus Bio-Medico University of Rome, Rome, Italy; ^2^ Translational Oncology Laboratory, Campus Bio-Medico University of Rome, Rome, Italy; ^3^ Pathology, Campus Bio-Medico University of Rome, Rome, Italy; ^4^ Anatomic Pathology, General Hospital of Treviso, Treviso, Italy; ^5^ Department of Surgical, Oncological and Oral Sciences, University of Palermo, Palermo, Italy; ^6^ Sbarro Institute for Cancer Research and Molecular Medicine, College of Science and Technology, Temple University BioLife Science, Philadelphia, PA, USA; ^7^ Department of Medicine, Surgery & Neuroscience University of Siena, Italy

**Keywords:** Dicer, Drosha, mir-155, liposarcoma

## Abstract

Liposarcoma (LPS) is the most common soft tissue sarcoma. It has been demonstrated that mir-155 was the most overexpressed miRNA in well-differentiated LPS(WDLPS)/dedifferentiated LPS (DDLPS). The aim of this study is to evaluate the involvement of Dicer, Drosha and mir-155 in development of LPS and their possible role in stratification of different histological subtypes. Dicer, Drosha and mir-155 mRNA levels were analyzed in formalin-fixed paraffin-embedded specimens from patients diagnosed with 62 LPS and compared with samples of adipose tissues of healthy donors. The experimental data were obtained using qRT-PCR comparing Dicer, Drosha and mir-155 expression levels in tumor samples versus normal fat. The tumor samples from LPS patients showed a significantly lower Dicer expression versus normal adipose tissue, while Drosha levels did not differ. Concerning mir155 expression levels, our results demonstrated a significant mir-155 up-regulation in all LPS subtypes versus normal adipose tissue except for WDLS. These findings demonstrate for the first time that Dicer is deregulated in LPS and show that mir-155 is differentially expressed in LPS subgroups and it could be a promising tool to improve LPS disease stratification and differential diagnosis.

## INTRODUCTION

Liposarcoma (LPS) is one of the most frequent sarcoma of adult representing 25% of soft tissue sarcomas. According to its clinic pathological and molecular genetic characteristics, it can be divided in four categories: well-differentiated LPS (WDLPS), dedifferentiated LPS (DDLPS), myxoid/round cell LPS (MLPS/RLPS) and pleomorphic LPS (PLPS) [1]. Classification in these subtypes represents the most important determinant of clinical behavior and outcome [2–6]; however, little is known about the genetic events that led to a specific LPS subtype. Moreover, a significant variability in predictive value exists among different clinical laboratories and hospitals with regard to the assessment of LPS histologic subtype, which has been primarily based on morphologic appearance of the tumor.

LPSs with similar morphologic appearance can follow different clinical courses and show divergent responses to systemic therapy. Particularly a correct histologic categorization is important for patients with PLPS that have a 3-fold higher risk of distant metastasis compared with patients with DDLPS [7]. Generally speaking, the accurate discrimination between different LPS histologic subtypes based on morphology alone is often a challenge even for an experienced soft tissue pathologist.

Nowadays immunohistochemical analysis has become an useful tool especially in order to better distinguish benign adipose tissue from LPS. It is well known that WDLPS and DDLPS present amplification of MDM2 and CDK4 genes on chromosome 12q13–15 as opposed to benign adipose tumors and other sarcoma subtypes. Thus MDM2 and CDK4 immunostaining are helpful adjuncts to differentiate WDLPS from benign adipose tumors and discriminate DDLPS from poorly differentiated sarcomas [8], but others molecular factors differentially expressed in LPS subgroups could represent an helpful tool to identify subtypes and predict disease outcome. In addition, identification of early genetic events in LPS pathogenesis could provide also a potential therapeutic target in a scenario characterized by only few effective treatment other than surgery.

Several studies showed that microRNA and its master regulators Dicer and Drosha are directly involved in proliferation, differentiation and apoptosis; their altered expression seems to play a key role in defining aggressive behavior and therefore the prognosis of different cancer subtypes [9–13]. A recent study focused on miRNA expression profile in human LPS and normal fat specimens showed that the oncogenic mir-155 was the most overexpressed miRNA in WD/DDLPS [14]. Furthermore, some data suggest that knockdown of mir-155 delayed tumor cell growth, decreased colony formation and induced G1-S cell cycle arrest *in vitro* and blocked tumor growth in murine xenografts *in vivo* [14].

Accordingly, the aim of this study was to examine whether Dicer, Drosha and mir-155 expression levels were deregulated in patients affected by LPS with particular regard to the accordance with histological diagnosis in different LPS subtypes (WDLPS, DDLPS, MLPS/RLPS and PLPS).

The data providing by this analysis and the enlargement of this series have the potential to improve LPS disease stratification and differential diagnosis.

## RESULTS

### *In silico* analyses

**Figure 1:**
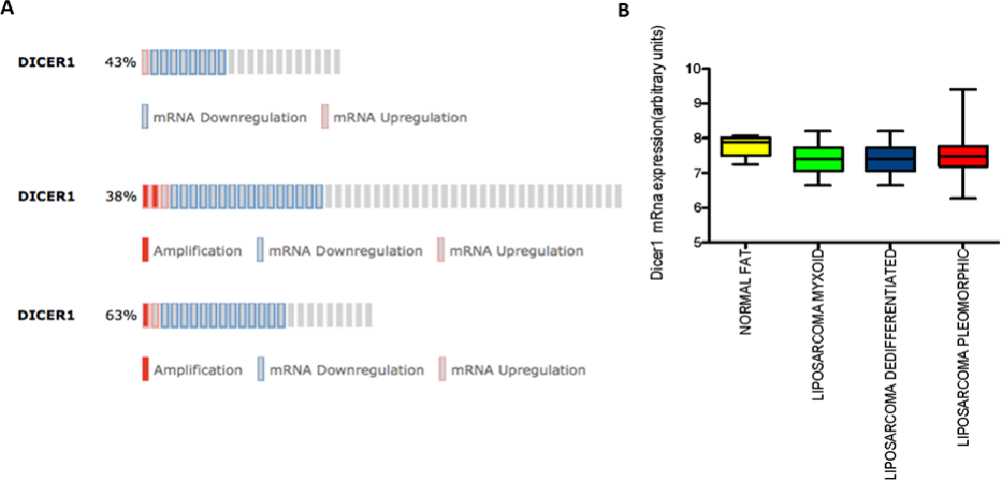
In silico analyses **(A)** Dicer expression in three different Case Sets: MLPS/RLPS (21 samples), Dicer altered in 9 (43%); DDLPS (50 samples), Dicer altered in 19 (38%); PLPS (24 samples), Dicer altered in 15 (63%). **(B)** Dicer mRNA levels in different LPS histologies compared to normal fat. (*p* value > 0.05).

In order to get preliminary information about Dicer expression in LPS, we analyzed the transcriptomic profiles of 21 MLPS/RLPS, 50 DDLPS, 24 PLPS and 9 normal fat (GSE 21124) searching for Dicer expression level and we found no significant changes in mRNA levels across the different histologies (Figure 1A and Figure 1B). Starting from these results we decided to perform a second analysis in our series using a more reliable and sensitive method such as qRT-PCR.

### Dicer and Drosha expression in LPS subtypes

To investigate if Dicer and Drosha expression was deregulated in LPS, we compared the mRNA levels of these two genes between cancer specimens and normal adipose tissue.

**Figure 2:**
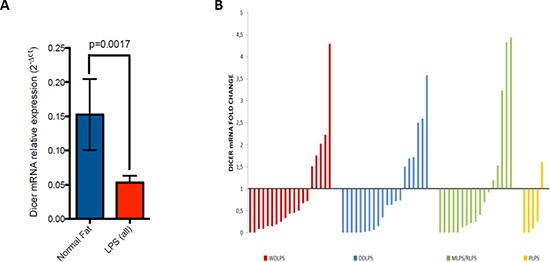
**(A)** Dicer mRNA expression in LPS histotypes compared to normal fat (*p* value = 0.0017). **(B)** Waterfall plot of Dicer mRNA levels in all LPS histotypes normalized with the median value of Dicer expression in normal fat

Considering LPS histotypes taken together we showed that Dicer mRNA levels were significantly decreased compared to the levels in normal tissue (*p* = 0.0017) (Figure 2A).

Subsequently, analyzing Dicer expression values in different LPS histotypes compared to normal fat (Figure 2B) we found a statistically significant reduction of Dicer mRNA levels in all specific subtypes except for WDLS (Table 1).

**Table 1:** Dicer expression values of different LPS histotype (median and mean) Mann–Whitney *U*-test was used to compare Dicer expression values among LPS subtypes and normal fat. In bold *p* value ≤ 0.05

	NORMAL FAT	WDLPS	DDLPS	MLPS\RLPS	PLPS
**Number of samples**	15	19	20	17	5
**Median**	0.06	0.026	0.02924	0.015	0.005
**Mean**	0.1525	0.05604	0.05051	0.06148	0.0232
***P* Value (vs Normal Fat)**		0.075	**0.017**	**0.021**	**0.0113**
***P* Value (vs WDLPS)**			0.662	0.6909	0.1642
***P* Value (vs DDLPS)**				0.9752	0.3906
***P* Value (vs MLPS/RLPS)**					0.4493

No statistically significant differences were obtained comparing the Dicer levels normalized to the housekeeping gene (PPIA) among different tumor histotypes albeit a median Dicer expression reduction was recorded in MLPS/RLPS and in PLPS (Table 1).

Regarding to Drosha expression levels did not present any statistically significant difference when compared to the normal adipose tissue values and among the LPS subtypes.

### Mir-155 expression in LPS subtypes

Mir-155 is one of the most relevant LPS related miRNA described in literature, indeed it is involved in tumor cell proliferation, migration, and invasion [14].

**Figure 3:**
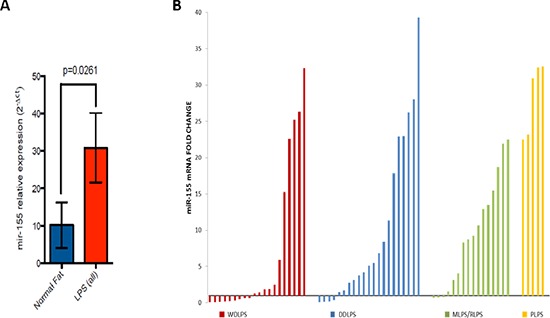
**(A)** mir-155 expression levels in LPS histotypes compared to normal fat (*p* value = 0.026). **(B)** Waterfall plot of mir-155 expression levels in all LPS histotypes normalized with the median value of mir-155 expression in normal fat

Our results showed a significant mir-155 up-regulation in all LPS subtypes taken together compared to normal fat (*p* = 0.0261) (Figure 3A).

Analyzing mir-155 expression levels in different histotypes we observed a significant up-regulation in DDLPS, MLPS and PLPS compared to normal tissue (Figure 3B; Table 2).

**Table 2:** Mir-155 expression values of different LPS histotype (median and mean) Mann–Whitney *U*-test was used to compare mir-155 expression values among LPS subtypes and normal fat. In bold *p* value ≤ 0.05

	NORMAL FAT	WDLPS	DDLPS	MLPS\RLPS	PLPS
**Number of samples**	17	20	21	16	5
**Median**	0.922	1.221	4.701	8.289	28.5
**Mean**	10.19	15.64	52.78	17.45	42.68
***P* Value (vs Normal Fat)**		0.8431	**0.0371**	**0.0081**	**0.0077**
***P* Value (vs WDLPS)**			**0.044**	**0.0316**	**0.0191**
***P* Value (vs DDLPS)**				0.963	0.103
***P* Value (vsMLPS/RLPS)**					**0.0431**

Intriguingly we found an increasing degree on mir-155 overexpression, normalized to the endogenous control RNU6B, from WDLPS to PLPS (low grade/high grade LPS respectively). Indeed in WDLPS mir-155 expression levels were significantly reduced compared to DDLPS, MLPS/RLPS and PLPS (Table 2). Moreover our results showed a statistically significant difference in mir-155 expression between DDLPS and PLPS subtypes (Table 2).

## DISCUSSION

Several studies have investigated the role of Dicer and Drosha in cancer tissues from different sites and their aberrant expression is commonly reported. Recently, it has been demonstrated that levels of Dicer could be used as prognostic markers in non-small cell lung cancer (NSCLC) [11] and in breast cancer patients where reduced mRNA expression is significantly associated with poor patient survival [15–16]. Moreover, Merritt et al. showed that levels of Dicer and Drosha are prognostic factors in patients with ovarian cancer [10]. Our group have been previously demonstrated that Dicer mRNA levels was independent predictors of favorable outcome and response in patients affected by advanced colorectal cancer treated with bevacizumab-based therapy [17].

Concerning soft tissue sarcoma, few data about Dicer and Drosha expression are available nowadays. In leiomiosarcoma (LMS) it has been demonstrated that higher Dicer levels correlated with a poorly differentiated phenotype suggesting its involvement in the progression of this neoplasm [13]. Another recent work showed how Dicer functions as a haploinsufficient tumor suppressor gene in pleomorphic sarcoma causing a loss of miRNA expression and leading to the development of distant metastases and to worse prognosis [18].

In LPS, one of the most frequent sarcoma of adult representing 25% of soft tissue sarcomas, no data about Dicer and Drosha expression are available, so far.

Due to this gap in our knowledge and in order to minimize biological heterogeneity between patients, we analyzed a group of LPS tumor samples and 15 benign samples of adipose tissues, as control group, to investigate if Dicer and Drosha mRNA levels were deregulated in LPS. The results showed that Dicer mRNA levels were significantly decreased in each LPS subtype compared to the levels in normal adipose tissue and this down-regulation was particularly evident in the PLPS samples, the most dedifferentiated subtypes.

Previously published studies show conflicting results about prognostic role of Dicer across tumour types. Reduced Dicer mRNA in cancers of the breast [16–17], lung [11], and ovary [10] was associated with aggressive phenotypic features, whereas the converse is described in prostatic [19], ovarian [20–21], esophageal [22] and colorectal [23] cancers by others and several reasons have been proposed for these discrepancies. It has been known that miRNA expression patterns are highly specific for cell type and cellular differentiation status. Thus, depending on whether the net effect of the majority of miRNAs in a given cell is oncogenic or tumor suppressive, the loss of Dicer expression can have opposite consequences on cell survival and proliferation. Thus, Dicer deregulation may be site specific and its role may differ in different tumors and in different subtypes.

Our data suggest a potential role of Dicer in the dedifferentiation process involving adipocyte cells from WDLPS to less differentiated high grade pleomorphic subtype. Indeed some evidences have been reported that Dicer regulates the expression of several genes involved in the first step of adipocyte differentiation through the regulation of some miRNA synthesis [24].

On the other hand, Drosha expression levels did not present any statistically significant difference when compared against the normal adipose tissue values and among the LPS types.

Starting from the evidences reported by Zhang [14] demonstrating mir-155 up-regulation in WDLPS/DDLPS, we analyzed mir-155 expression levels in tumor samples and normal fat. Our findings not only confirm a significant up-regulation of mir-155 compared to normal tissue in DDLPS, but also highlight a more pronounced increase of mir-155 levels in MLPS and PLPS. These data clearly demonstrate the involvement of mir-155 in LPS progression and suggest its possible role as biomarker for discrimination between different subtype of LPS.

In conclusion we demonstrated for the first time that Dicer is deregulated in LPS and its decrease seems to be associated with more aggressive histological subtype. Moreover we showed that mir-155 is differentially expressed in LPS subgroups and it could be a promising tool to provide improvements in disease stratification, differential diagnosis and predict disease outcome.

## MATERIALS AND METHODS

### Microarray dataset analyses

Microarray data and clinical information were extracted from GEO (dataset accession number: GSE21124). 206061_s_at probe (Affymetrix Human Genome U133A Array) was used for Dicer expression analysis.

### Patients and samples

In our study we retrospectively included 61 patients diagnosed with LPS seen at Campus Bio-Medico University (UCBM) of Rome and at “S. Maria di Ca' Foncello” Hospital of Treviso, as the reference center for Pathology of the Italian Rare Cancer Network. These patients were histologically classified as follow: WDLPS (19 pts), DDLPS (20 pts), MLPS (17 pts), and PLPS (6 pts). For all patients formalin-fixed paraffin-embedded (FFPE) surgical specimens collected prior to start of any therapy were available.

In addition, we collected 15 benign samples of adipose tissues (control group) obtained from Pathology Division of UCBM. The study was evaluated by the Local Ethics Committee of UCBM with a positive outcome for the use of all samples, without a written consent, as stated by the Guarantor for the protection of personal data (general authorization of personal data management for scientific purposes published on March 1st 2012). Based on all this evaluation the local ethic committee approved the study with the request of anonymize patients records requested by the Guarantor.

### Quantitative real-time polymerase chain reaction (qRT-PCR)

FFPE sections were treated with xylene to remove paraffin; the tissue was subsequentially incubated overnight at 56°C with Proteinase K (Qiagen, UK) to allow samples lysis. Total RNA was extracted using the Trizol reagent (Invitrogen, CA, USA) according to the manufacturer's instructions. RNA was treated with DNase (DNAse Turbo, Applied Biosystems, CA, USA) to avoid genomic DNA contamination. The concentration and purity of the isolated RNA (A260/A280 ratio between 1.8 and 2.0 were accepted) were measured using a NanoDrop ND-1000 Spectrophotometer (Thermo Fisher Scientific, DE, USA). cDNA was synthetized using the High Capacity cDNA Reverse Transcription Kit (Applied Biosystems, CA, USA) according to the manufacturer's recommendations. mRNA levels were measured by qRT-PCR performed on a 7900HT Fast Real-Time PCR System (Applied Biosystems, CA, USA). In all samples, inventoried TaqMan Gene Expression Assays for Dicer (Hs00229023_m1), Drosha (Hs00203008_m1), PPIA (Hs99999904_m1) [25], mir-155 (002623) and mir-RNU6B (001093) were used. For each samples, genes and miRNA expression levels were normalized to house-keeping control gene/miRNA (PPIA and mir-RNU6B respectively) through the 2^−deltaCt^ calculation. Three technical replicates of all samples and ddH_2_O, as non-template control, were performed and analyzed for every reaction mix. PCR cycling included the following steps: 1 cycle at 95°C for 10 min, 45 times at 95°C for 15s and 60°C for 1 min.

### Statistical analysis

Relative fold-changes were obtained by normalizing the Dicer, Drosha mRNA and mir-155 expression levels in pathological samples to the relative amount in healthy samples using the 2^−ΔΔCt^ method (ABI software, Applied Biosystems, CA, USA). Statistical analysis were performed using GraphPad Prism (version 6.01; GraphPad Software); Mann–Whitney *U*-test was used to compare different groups and *p*-values ≤ 0.05 were considered statistically significant.

## References

[1] Fletcher CDM, Rydholm A, Singer S, Sundaram M, Coindre JM, Fletcher CDM, Unni KK, Mertens F (2002). Soft tissue tumours: Epidemiology, clinical features, histopathological typing and grading. World Health Organization classification of tumours. Pathology and genetics of tumours of soft tissue and bone.

[2] Antonescu CR, Tschernyavsky SJ, Decuseara R, Leung DH, Woodruff JM, Brennan MF, Bridge JA, Neff JR, Goldblum JR, Ladanyi M (2001). Prognostic impact of P53 status, TLS-CHOP fusion transcript structure, and histological grade in myxoid liposarcoma: a molecular and clinicopathologic study of 82 cases. Clin Cancer Res.

[3] Eilber FC, Eilber FR, Eckardt J, Rosen G, Riedel E, Maki RG, Brennan MF, Singer S (2004). The impact of chemotherapy on the survival of patients with high-grade primary extremity liposarcoma. Ann Surg.

[4] Gebhard S, Coindre JM, Michels JJ, Terrier P, Bertrand G, Trassard M, Taylor S, Château MC, Marquès B, Picot V, Guillou L (2002). Pleomorphic liposarcoma: clinicopathologic, immunohistochemical, and follow-up analysis of 63 cases: a study from the French Federation of Cancer Centers Sarcoma Group. Am J Surg Pathol.

[5] Kooby DA, Antonescu CR, Brennan MF, Singer S (2004). Atypical lipomatous tumor/well-differentiated liposarcoma of the extremity and trunk wall: importance of histological subtype with treatment recommendations. Ann Surg Oncol.

[6] Singer S, Antonescu CR, Riedel E, Brennan MF (2003). Histologic subtype and margin of resection predict pattern of recurrence and survival for retroperitoneal liposarcoma. Ann Surg.

[7] Singer S, Socci ND, Ambrosini G, Sambol E, Decarolis P, Wu Y, O'Connor R, Maki R, Viale A, Sander C, Schwartz GK, Antonescu CR (2007). Gene expression profiling of liposarcoma identifies distinct biological types/subtypes and potential therapeutic targets in well-differentiated and dedifferentiated liposarcoma. Cancer Res.

[8] Binh MB, Sastre-Garau X, Guillou L, de Pinieux G, Terrier P, Lagacé R, Aurias A, Hostein I, Coindre JM (2005). MDM2 and CDK4 immunostainings are useful adjuncts in diagnosing well-differentiated and dedifferentiated liposarcoma subtypes: a comparative analysis of 559 soft tissue neoplasms with genetic data. Am J Surg Pathol.

[9] Caldas SSEaMC (2008). MicroRNA:implications for cancer. Virchows Arch.

[10] Merritt WM, Lin YG, Han LY, Kamat AA, Spannuth WA, Schmandt R, Urbauer D, Pennacchio LA, Cheng JF, Nick AM, Deavers MT, Mourad-Zeidan A, Wang H (2008). Dicer, Drosha, and outcomes in patients with ovarian cancer. N Engl J Med.

[11] Karube Y, Tanaka H, Osada H, Tomida S, Tatematsu Y, Yanagisawa K, Yatabe Y, Takamizawa J, Miyoshi S, Mitsudomi T, Takahashi T (2005). Reduced expression of Dicer associated with poor prognosis in lung cancer patients. Cancer Sci.

[12] Papachristou DJ, Rao UN, Korpetinou A, Giannopoulou E, Sklirou E, Kontogeorgakos V, Kalofonos HP (2012). Prognostic significance of Dicer cellular levels in soft tissue sarcomas. Cancer Invest.

[13] Papachristou DJ, Sklirou E, Corradi D, Grassani C, Kontogeorgakos V, Rao UN (2012). Immunohistochemical analysis of the endoribonucleases Drosha, Dicer and Ago2 in smooth muscle tumours of soft tissues. Histopathology.

[14] Zhang P, Bill K, Liu J, Young E, Peng T, Bolshakov S, Hoffman A, Song Y, Demicco EG, Terrada DL, Creighton CJ, Anderson ML, Lazar AJ (2012). MiR-155 is a liposarcoma oncogene that targets casein kinase-1α and enhances β-catenin signaling. Cancer Res.

[15] Grelier G, Voirin N, Ay AS, Cox DG, Chabaud S, Treilleux I, Léon-Goddard S, Rimokh R, Mikaelian I, Venoux C, Puisieux A, Lasset C, Moyret-Lalle C (2009). Prognostic value of Dicer expression in human breast cancers and association with the mesenchymal phenotype. Br J Cancer.

[16] Dedes KJ, Natrajan R, Lambros MB, Geyer FC, Lopez-Garcia MA, Savage K, Jones RL, Reis-Filho JS (2011). Down-regulation of the miRNA master regulators Drosha and Dicer is associated with specific subgroups of breast cancer. Eur J Cancer.

[17] Vincenzi B, Zoccoli A, Schiavon G, Iuliani M, Pantano F, Dell'aquila E, Ratta R, Muda AO, Perrone G, Brunelli C, Correale P, Riva E, Russo A (2013). Dicer and Drosha expression and response to Bevacizumab-based therapy in advanced colorectal cancer patients. Eur J Cancer.

[18] Mito JK, Min HD, Ma Y, Carter JE, Brigman BE, Dodd L, Dankort D, McMahon M, Kirsch DG (2013). Oncogene-dependent control of miRNA biogenesis and metastatic progression in a model of undifferentiated pleomorphic sarcoma. J Pathol.

[19] Chiosea S, Jelezcova E, Chandran U, Acquafondata M, McHale T, Sobol RW, Dhir R (2006). Up-regulation of dicer, a component of the MicroRNA machinery, in prostate adenocarcinoma. Am J Pathol.

[20] Flavin RJ, Smyth PC, Finn SP, Laios A, O'Toole SA, Barrett C, Ring M, Denning KM, Li J, Aherne ST, Aziz NA, Alhadi A, Sheppard BL (2008). Altered eIF6 and Dicer expression is associated with clinicopathological features in ovarian serous carcinoma patients. Mod Pathol.

[21] Vaksman O, Hetland TE, Trope' CG, Reich R, Davidson B (2012). Argonaute, Dicer, and Drosha are up-regulated along tumor progression in serous ovarian carcinoma. Hum Pathol.

[22] Sugito N, Ishiguro H, Kuwabara Y, Kimura M, Mitsui A, Kurehara H, Ando T, Mori R, Takashima N, Ogawa R, Fujii Y (2006). RNASEN regulates cell proliferation and affects survival in esophageal cancer patients. Clin Cancer Res.

[23] Faber C, Horst D, Hlubek F, Kirchner T (2011). Overexpression of Dicer predicts poor survival in colorectal cancer. Eur J Cancer.

[24] Fujimoto Y, Nakagawa Y, Shingyouchi A, Tokushige N, Nakanishi N, Satoh A, Matsuzaka T, Ishii KA, Iwasaki H, Kobayashi K, Yatoh S, Suzuki H, Yahagi N (2012). Dicer has a crucial role in the early stage of adipocyte differentiation, but not in lipid synthesis, in 3T3-L1 cells. Biochem Biophys Res Commun.

[25] Neville MJ, Collins JM, Gloyn AL, McCarthy MI, Karpe F (2011). Comprehensive human adipose tissue mRNA and microRNA endogenous control selection for quantitative real-time-PCR normalization. Obesity (Silver Spring).

